# Line breaks in subtitling: an eye tracking study on viewer preferences

**DOI:** 10.16910/jemr.11.3.2

**Published:** 2018-05-17

**Authors:** Olivia Gerber-Morón, Agnieszka Szarkowska

**Affiliations:** Universitat Autònoma de Barcelona, Spain; University College London, UK; University of Warsaw, Poland

**Keywords:** Eye movements, eye tracking, reading, subtitling, line breaks, individual differences, segmentation, audiovisual translation, syntactic processing

## Abstract

There is a discrepancy between professional subtitling guidelines and how they are implemented in real life. One example of such discrepancy are line breaks: the way the text is divided between the two lines in a subtitle. Although we know from the guidelines how subtitles *should* look like and from watching subtitled materials how they *really* look like, little is known about what line breaks viewers would prefer. We examined individual differences in syntactic processing and viewers’ preferences regarding line breaks in various linguistic units, including noun, verb and adjective phrases. We studied people’s eye movements while they were reading pictures with subtitles. We also investigated whether these preferences are affected by hearing status and previous experience with subtitling. Viewers were shown 30 pairs of screenshots with syntactically segmented and non-syntactically segmented subtitles and they were asked to choose which subtitle in each pair was better. We tested 21 English, 26 Spanish and 21 Polish hearing people, and 19 hard of hearing and deaf people from the UK. Our results show that viewers prefer syntactically segmented line breaks. Eye tracking results indicate that linguistic units are processed differently depending on the linguistic category and the viewers’ profile.

## Introduction

It is a truth universally acknowledged that subtitles should be easy to read and not stand in viewers’ enjoyment of a film. One way of enhancing subtitle readability is segmentation, i.e. the way the text is divided between the two lines in a subtitle. Both subtitling scholars and professionals believe that subtitle segmentation should follow syntactic rules (1, 2, 3, 4, 5, 6, 7, 8). This means that linguistic units should be kept together in one line. For instance, rather than having a subtitle segmented in this way (2):

We are aiming to get a 

better television service.

a well-segmented subtitle would have the indefinite article ‘*a*’ in the second line together with the rest of the noun phrase it belongs to:

We are aiming to get

a better television service.

As subtitles compete for screen space and viewers’ attention with images, good subtitle segmentation is crucial to optimise readability and to enhance viewers’ enjoyment of the film (3). In this study, we look into viewers’ preferences on subtitle segmentation and its impact on readability.

### Syntactically-cued text and reading

When reading, people make sense of words by grouping them into phrases – a process known as parsing (9). Parsing is done incrementally, word by word: readers do not wait until the end of the sentence to interpret it, but try to make sense of it while they are reading (10, 11). To understand a sentence, readers must “first identify its syntactic relations” (11). If text is not syntactically cued, the reader’s comprehension may be disrupted. Syntactic ambiguities leading the reader to an incorrect interpretation, known as “garden path” sentences, need to be reanalysed and disambiguated (11, 12). These ambiguities and disruptions affect eye movements, as readers make longer fixations and regress to earlier parts of the sentence to disambiguate unclear text (10).

Previous studies on reading printed text showed that syntactically-cued text facilitates reading (13, 14, 15), resulting in fewer dysfluencies at line breaks than uncued texts (13). Dividing phrases based on syntactic units has also been found to improve children’s reading comprehension (14, 15). From previous eye tracking literature, we know that some grammatical structures are more difficult to process than others, resulting in regressive eye movements and longer reading times (16, 17, 18). In this study, we expect to find eye movement disfluencies (revisits, longer dwell time) in non-syntactically segmented text.

### Linguistic units in subtitle segmentation

Subtitling guidelines recommend that subtitle text should be presented in sense blocks and divided based on linguistic units (1, 19, 20, 21), at the highest syntactic nodes possible (6). At the phrase level, it is believed (7) that the following phrases should be displayed on the same subtitle line: noun phrases (nouns preceded by an article); prepositional phrases (simple and/or complex preposition heading a noun or noun phrase); and verb phrases (auxiliaries and main verbs or phrasal verbs). At the clause and sentence level, constructions that should be kept on the same subtitle line include (7): coordination constructions (sentential conjunctions such as ‘and’ and negative constructions with ‘not’); subordination constructions (clauses introduced by the conjunction ‘that’); *if*-structures and comparative constructions (clauses preceded by the conjunction ‘than’).

Similar rules regarding line breaks are put forward in many subtitling guidelines endorsed by television broadcasters and media regulators (2, 8 22, 23, 24, 25). According to them, the parts of speech that should not be split across a two-line subtitle are: article and noun; noun and adjective; first and last name; preposition and following phrase; conjunction and following phrase/clause; prepositional verb and preposition; pronoun and verb; and parts of a complex verb. However, when there is a conflict, synchronisation with the soundtrack should take precedence over line breaks (2).

### Geometry in subtitle segmentation

Apart from sense blocks and syntactic phrases, another important consideration in how to form a two-line subtitle is its geometry (1, 3, 5, 6). When watching subtitled videos, viewers may not be aware of syntactic rules used to split linguistic units between the lines. What they may notice instead is subtitle shape: either closer to a pyramid or trapezoid with one line shorter than the other, or a rectangle with two lines of roughly equal length.

It is generally believed that lines within a subtitle should be proportionally equal in length because “untidy formats are disliked by viewers” (1) and people are used to reading printed material in a rectangular format (6). When two lines of unequal length are used, “the upper line should preferably be shorter to keep as much of the image as free” (19). If geometry is in conflict with syntax, then preference is given to the latter (6).

In view of the above, it is plausible that viewers make their preferences based on the shape rather than syntax (1, 26). Tests with viewers are therefore needed to understand subtitle segmentation preferences and to establish the effects of line breaks on subtitling processing.

### Empirical studies on subtitle segmentation

Previous research on subtitle segmentation, including studies with eye tracking, has been limited and inconclusive. In a study on the cognitive effectiveness of subtitle processing (27), no differences were found in processing subtitles with and without syntactic-based segmentation, except for longer fixations in non-syntactically segmented text. Similarly, Gerber-Morón and Szarkowska (28) did not find differences in comprehension between syntactically and non-syntactically segmented subtitles, but reported higher cognitive load in the latter. In contrast, a study on text chunking in live subtitles (29) showed that subtitles segmented following linguistic phrases facilitate subtitle processing. They found a significant difference in the number of eye movements between the subtitles and the image compared to non-syntactically segmented subtitles displayed word by word.

### Different types of viewers

People may watch subtitled films differently depending on whether or not they are familiar with subtitling. Yet, despite an increasingly growing number of eye tracking studies on subtitling (30, 31, 32, 33), little is known about the role of viewers’ previous experience with subtitling on the way they process subtitled videos. Perego et al. (34) conducted a cross-national study on subtitle reception and found that Italians, who are not habitual subtitle users, spent most of the watching time on reading subtitles and took more effort processing subtitles. In a study on eye movements of adults and children while reading television subtitles (35), longer fixations in the text were observed in children, who were less experienced in subtitling than adults. Similar fixation durations were obtained in another study on the processing of native and foreign language subtitles in native English speakers (30), which was attributed to the lack of familiarity with subtitles.

Apart from previous experience with subtitling, another factor that impacts on the processing of subtitled videos is hearing status (36). Burnham et al. (37) note that “hearing status and literacy tend to covary” (p. 392). Early deafness has been found to be a predictor of poor reading (38, 39, 40, 41, 42, 43, 44). In consequence, deaf viewers may experience difficulties when reading subtitles and their comprehension of subtitled content may be lower than that of hearing viewers (45, 46, 47). One of the difficulties experienced by deaf people when reading is related to definite and indefinite articles (48, 49). Deaf people spend more time reading function words in subtitles (such as determiners, prepositions, conjunctions or auxiliary verbs) than hard of hearing and hearing viewers (50). This has been attributed to the fact that many function words do not exist in sign languages, that such words tend to be short and unstressed, and therefore more difficult to identify, and that they have “low fixed semantic content outside of specific context in which they occur” (48). Given that function words are an important part of the linguistic units split between the two subtitle lines, in this study we investigate whether hearing status and previous experience with subtitling affects the preferences for or against syntactically-cued text.

### Different types of viewers

This study adopts the viewers’ perspective on subtitle segmentation by analysing people’s preferences and reactions to different types of line breaks. To investigate these issues, the approach we developed was three-fold. First, we examined the preferences of different groups of subtitle viewers with the goal of identifying any potential differences depending on their experience with subtitling, their hearing status and the nature of the linguistic units. Second, we analysed viewers’ eye movements while they were reading syntactically segmented and non-syntactically segmented subtitles. Drawing on the assumption that processing takes longer in the case of more effortful texts (51), we predicted that syntactically segmented text would be preferred by viewers, whereas non-syntactically segmented text would take more time to read and result in higher mean fixation durations, particularly in the case of viewers less experienced with subtitling or deaf, given their known difficulties with processing syntactic structures (52, 53, 54, 55, 56, 57). Finally, we invited participants to a short semi-structured interview to elicit their views on subtitle segmentation.

This study consists of two experiments: in Experiment 1 we tested hearing viewers from the UK, Poland, and Spain, while in Experiment 2 we tested British deaf, hard of hearing and hearing people. In each experiment, participants were asked to choose subtitles which they thought were better from 30 pairs of screenshots (see the Methods section). In each pair, one subtitle was segmented following the established subtitling rules, as described in the Introduction, and the other violated them, splitting linguistic units between the two lines. After the experiment, participants were also asked whether they made their choices based on linguistic considerations or rather on subtitle shape.

Using a mixed-methods approach, where we combined preferences, eye tracking and interviews, has enabled us to gain unique insights into the reception of subtitle segmentation among different groups of viewers. To the best of our knowledge, no previous research has been conducted into viewers’ preferences on subtitle segmentation, using such a wide selection of linguistic units. The results of this study are particularly relevant in the context of current subtitling practices and subtitle readability.

## Methods

The study took place at University College London. Two experiments were conducted, using the same methodology and materials. The study received full ethical approval from the UCL Research Ethics Committee.

### Participants

Experiment 1 involved 68 participants (21 English, 21 Polish, and 26 Spanish native speakers) ranging from 19 to 42 years of age (*M*=26.51, *SD*=6.02). Spanish speakers were included given their exposure to dubbing. Polish speakers were more accustomed to watching subtitles in comparison with Spanish speakers. English speakers were used as a control group. However, even though the participants came from different audiovisual translation traditions, most of them declared that subtitling is their preferred type of watching foreign films. They said they either use subtitles in their mother tongue or in English, which is not surprising given that the majority of the productions they watch are in English. This can be on the one hand be explained by changing viewers habits (58) and on the other by the fact that our participants were living in the UK. The fact that they are frequent subtitle users also makes them a good group to ask about certain solutions used in subtitles, such as line breaks.

As the subtitles in this study were in English, we asked Polish and Spanish participants to evaluate their proficiency in reading English using the Common European Framework of Reference for Languages (from A1 to C2). All the participants declared a reading level equal or higher than B1. Of the total sample of Polish participants, 3 had a C1 level and 18 had a C2 level. In the sample of Spanish participants, 1 had a B1 level, 4 had a B2 level, 5 had a C1 and 16 had a C2 level. No statistically significant differences were found between the proficiency of Polish and Spanish participants, *χ*^*2*^(3)=5.144, *p*=.162.

Experiment 2 involved either hearing, hard of hearing, or deaf participants from the UK. We recruited 40 participants (21 hearing, 10 hard of hearing and 9 deaf) ranging from 20 to 74 years of age (*M*=35.59, *SD*=13.7). Before taking part in the experiment, hard of hearing and deaf participants completed a demographic questionnaire with information on their hearing impairment, age of hearing loss onset, communication preferences, etc. and were asked if they described themselves as either deaf or hard of hearing. Of the total sample of deaf and hard of hearing participants, 10 were profoundly deaf, 6 were severely deaf and 3 had a moderate hearing loss. In relation to the age of onset, 7 were born deaf or hard of hearing, 4 lost hearing under the age of 8, 2 lost hearing between the ages of 9-17, and 6 lost hearing between the ages of 18-40. Except for two participants who used a BSL interpreter, other hard of hearing and deaf participants chose spoken and written English to communicate during the experiment.

Participants were recruited using the UCL Psychology pool of volunteers, social media (Facebook page of the SURE project, Twitter), and personal networking. Hard of hearing and deaf participants were recruited with the help of the National Association of Deafened People and the UCL Deafness, Cognition and Language Centre participant pool. Hearing participants were paid £10 for participating in the experiment, following UCL hourly rates for experimental participants. Hard of hearing and deaf participants received £25 in recognition of the greater difficulty in recruiting special populations.

### Design

In each experiment, we employed a mixed factorial design. The independent between-subject variables were language in Experiment 1 (English, Polish, Spanish) or hearing loss in Experiment 2 (hearing, hard of hearing and deaf), and the type of segmentation (syntactically segmented subtitles vs. non-syntactically segmented subtitles, henceforth referred to as SS and NSS, respectively). The main dependent variables were preferences on line breaks (SS and NSS) and eye tracking measures (dwell time, mean fixation duration and revisits).

### Materials

The subtitles used in this study were in English. One reason for this choice was that it would be difficult to test line breaks and subtitle segmentation across different languages. For instance, as opposed to English and Spanish, the Polish language does not have articles, so it would be impossible to compare this linguistic unit across the languages of study participants. Another reason for using English subtitles was that it is particularly in intralingual English-to-English subtitles on television in the UK (where our study materials came from and there this study was based) that non-syntactic based segmentation is common despite the current subtitling guidelines (2, 8).

**Figure 1.**
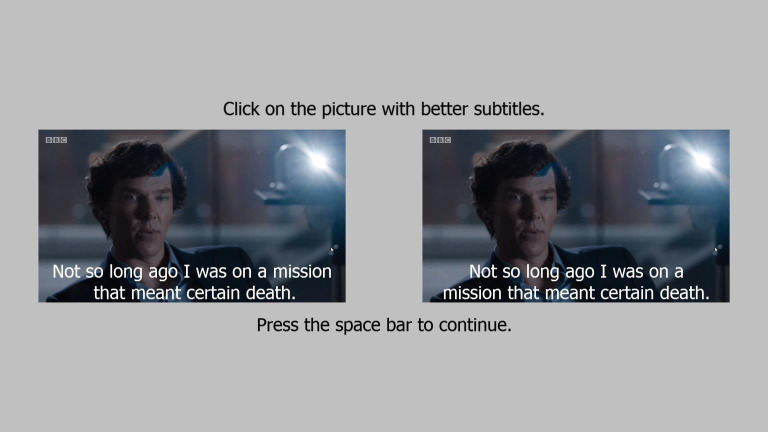
Stimulus example with syntactically segmented (left) and non-syntactically segmented text (right).on

The stimuli were 30 pairs of screenshots with subtitles in English from the BBC’s *Sherlock, Series 4 *(2017, dir. Mark Gatiss and Steven Moffat). Each pair contained exactly the same text, but differently segmented lines (see Figure 1). In one version, the two lines were segmented in accordance to subtitling standards, using syntactic rules to keep linguistic units on a single line (SS version). In the other version, syntactic rules were not followed and linguistic units were split between the first and the second line of the subtitle (NSS version).

The following ten categories of the most common linguistic units (59) were manipulated in the study:

1. Indefinite article + noun (*IndArt*) 

 2. Definite article + noun (*DefArt*) 

 3. To + infinitive (*ToInf*) 

 4. Compound (*Comp*) 

 5. Auxiliary + lexical verb (*AuxVerb*) 

 6. Sentence + sentence (*SentSent*) 

 7. Preposition (*Prep*) 

 8. Possessive (*Poss*) 

 9. Adjective + noun (*AdjN*) 

 10. Conjunction (*Conj*) 

For each of these categories, three instances, i.e. three different sentence stimuli, were shown (see Table 1 for examples). The presentation of screenshots (right/left) was counterbalanced, with 15 sentences in the SS condition displayed on the left, and 15 on the right. The order of presentation of the pairs (and therefore of different linguistic units) was randomised using SMI Experiment Centre.

**Table 1.**
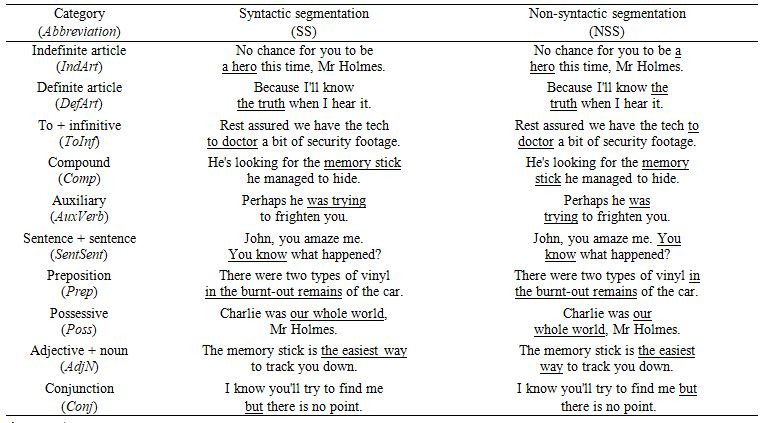
Examples of linguistic units manipulated in the syntactically segmented and non-syntactically segmented versions.

### Apparatus

SMI Red 250 mobile eye tracker was used with a two-screen set-up, one for experimenter and the other for the participant. Participants’ eye movements were recorded with the sampling rate of 250Hz. The minimum duration of a fixation was set at 80 ms. We used the SMI velocity-based saccade detection algorithm. Participants with tracking ratio below 80% were excluded from eye tracking analyses. The experiment was designed and conducted using the SMI Experiment Suite. SMI BeGaze and SPSS v. 24 were used to analyse the data.

### Dependent variables

**Figure 2.**
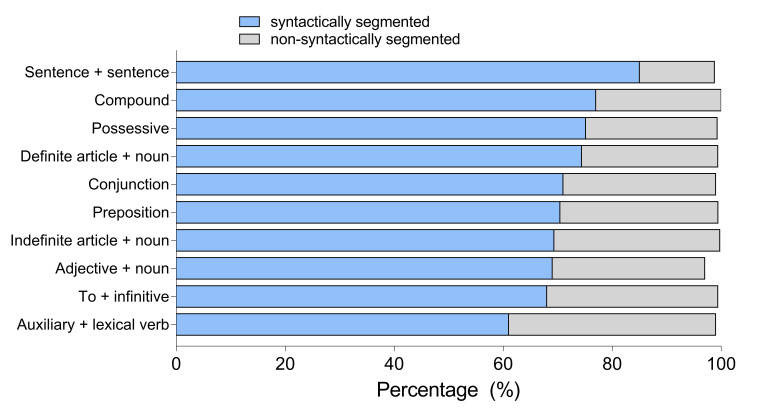
Visualisation of mouse clicks on syntactically segmented (left) and non-syntactically segmented (right) subtitles (SentSent condition).

The dependent variables were the preference score and three eye tracking measures (see Table 2). The preference score was calculated based on the preference expressed by a participant regarding each linguistic unit: as a percentage of people preferring SS or NSS subtitles in each linguistic unit. As there were three examples per unit, their scores were averaged per participant per unit. Participants expressed their preference by clicking on the picture with subtitles they thought were better (see Figure 2.)

After completing the test with 30 pairs of subtitles, participants were asked a multiple-choice follow-up question displayed on the screen:* What was most important for you when deciding which subtitles were better*? The following options were provided: *I chose those that looked like a pyramid/trapeze (shape), I chose those that looked like a rectangle (shape), I chose those that had semantic and syntactic phrases together, I don’t know*. In the post-test interview, we asked the participants if they prefer to have the first line in the subtitle shorter, longer or the same length as the second line, which prompted them to elaborate on their choices and allowed us to elicit their views on line breaks in subtitling.

Eye tracking analyses were conducted on data from areas of interest (AOIs) drawn for each subtitle in each screenshot. The three eye tracking measures used in this study are described in Table 2.

**Table 2.**
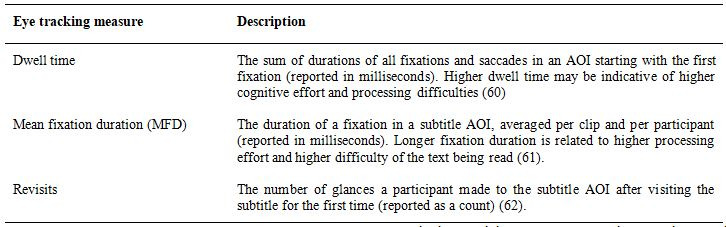
Description of the eye tracking measures.

### Procedure

Participants were tested individually in a lab. They were informed the study was on the quality of subtitles. The details of the experiment were not revealed until the end of the test during the debrief.

Before starting the test, participants read the information sheet, signed an informed consent form and underwent a 9-point calibration procedure. Participants saw 30 pairs of screenshots in randomised order. From each pair, participants had to select (i.e. click on) the screenshot with the subtitle segmentation they preferred (SS or NSS). Participants then answered the question on segmentation style preference. At the end, they undertook a short interview in which they expressed their views on subtitle segmentation based on the test and their personal experience with subtitles. The experiment concluded with the debrief of the study. The experiment lasted approximately 15 minutes, depending on the time it took the participants to answer the questions and participate in the interview.

## Results

All raw data, results and experimental protocols from this experiment are openly availably in RepOD repository (63).

### Experiment 1

### Preferences

We conducted a 2 x 3 mixed ANOVA with segmentation (SS vs. NSS subtitles) as a within-subjects factor and language (English, Polish, Spanish) as a between-subjects factor with a percentage of preference for a particular linguistic unit as a dependent variable. In all linguistic parameters tested, we found a large main effect of segmentation (see Table 3). The SS subtitles were preferred over the NSS ones.

**Figure 3.**
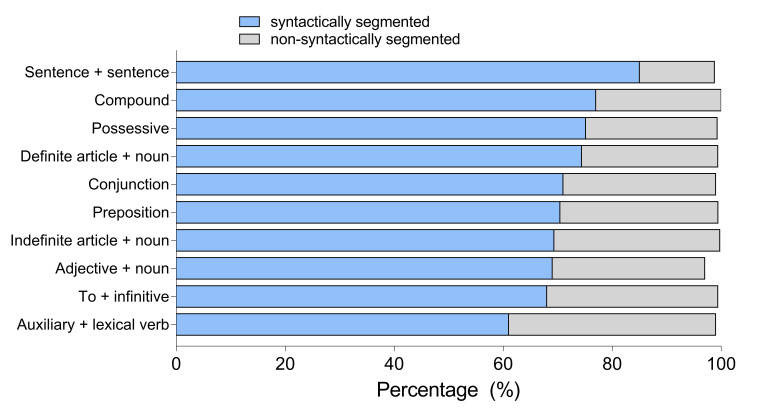
Preferences for SS and NSS subtitles by linguistic units in Experiment 1

Figure 3 shows preferences by linguistic units and Table 3 by participant groups. There were no differences between groups in any of the linguistic conditions and no interactions. This means that regardless of their mother tongue, all participants had similar preferences.

**Table 3.**
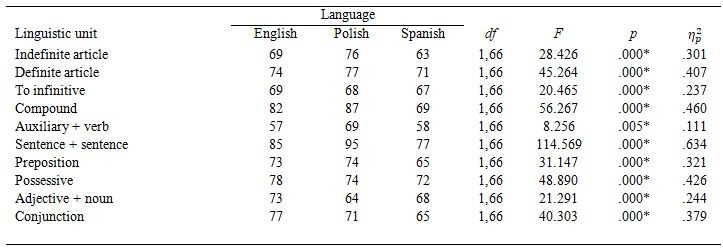
Percentage of participants who preferred the syntactically segmented condition.

**Figure 4.**
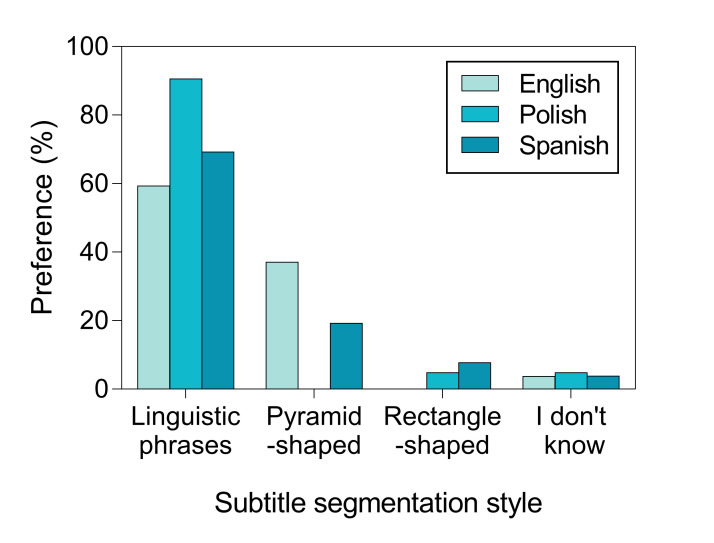
Segmentation preferences by group and style

As shown by Figure 4, the overwhelming majority of participants made their choices based on semantic and syntactic units rather than subtitle shape. Most Polish participants declared to prioritize semantic and syntactic units, whereas for English and Spanish participants pyramid shape was also considered as a choice.

### Eye tracking measures

Due to data quality issues, eye tracking analyses in Experiment 1 were conducted on 16 English, 16 Polish and 18 Spanish participants.

#### Dwell time

There was a main effect of segmentation on dwell time in all linguistic units apart from *ToInf*, *SentSent* and *Prep* (see Table 4). Dwell time was higher in most SS noun phrases (*IndArt*, *DefArt*, *Comp*, *Poss*) as well as in SS *Conj*, and lower in NSS *AuxVerb* and *AdjN*. There was no main effect of language on dwell time in any of the linguistic units. We found an interaction, approaching statistical significance, between segmentation and language in *Poss*, *F*(2,47)=3.092, *p*=.055, η_p_²=.116. We decomposed this interaction with simple effects with Bonferroni correction and found that for English participants there was a main effect of segmentation on dwell time in *Poss* , *F*(1,15)=13.217, *p*=.002, η_p_²=.468. Their dwell time was higher in the SS condition than in the NSS condition. There was no main effect for either Polish or Spanish participants.

**Table 4.**
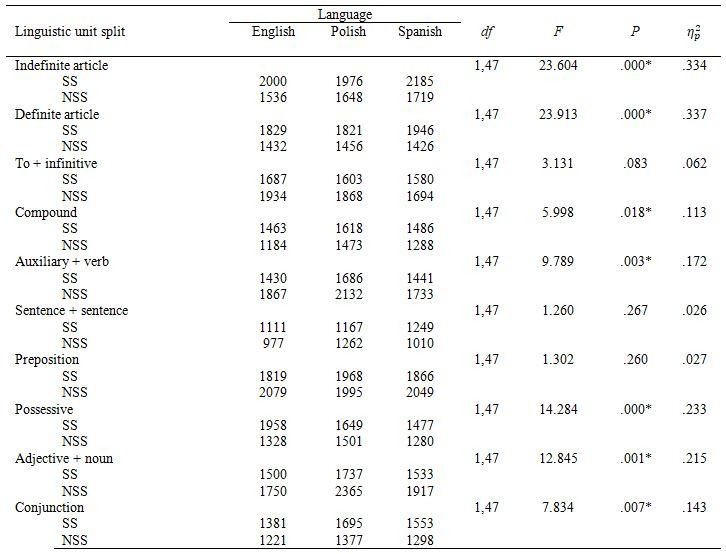
Dwell Time on subtitles by linguistic unit and segmentation (ms).

#### Mean fixation duration (MFD)

There was a main effect of segmentation on MFD only in one linguistic unit: *AdjN* (Table 5), where the SS condition resulted in higher MFD than the NSS one. We also found an interaction between segmentation and language in *DefArt*, *F*(2,41)=3.199, *p*=.051, η_p_²=.135. We decomposed this interaction with simple effects with Bonferroni correction and found that for Polish participants there was a main effect of segmentation on MFD in *DefArt*, *F*(1,12)=8.215, *p*=.014, η_p_²=.140, their mean fixation duration was longer for the NSS condition. There was no main effect for English or Spanish participants.

There was a main effect of language on MFD in a number of linguistic units (see Table 6). Post-hoc Bonferroni tests showed that Polish had significantly shorter MFD than Spanish participants in *IndArt*, *p*=.042, 95% CI [-74.52, -1.06]; *DefArt*, *p*=.020, 95% CI [-60.83, -4.21]; *ToInf*, *p*=.009, 95% CI [-68.47, -7.97]; *Comp*, *p*=.029, 95% CI [-61.92, -2.62]; and *Prep*, *p*=.034, 95% CI [-1.95, -66.18]. English participants did not differ from Polish or Spanish participants.**

**Table 5.**
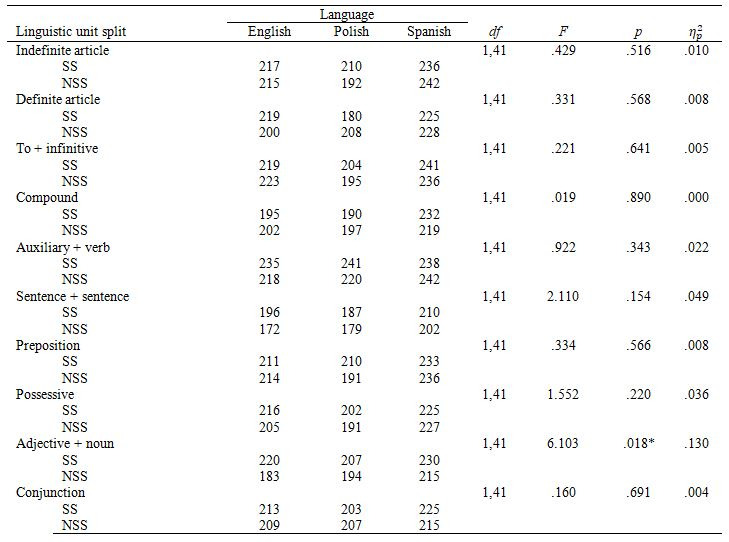
Mean fixation duration by linguistic unit and segmentation.

**Table 6.**
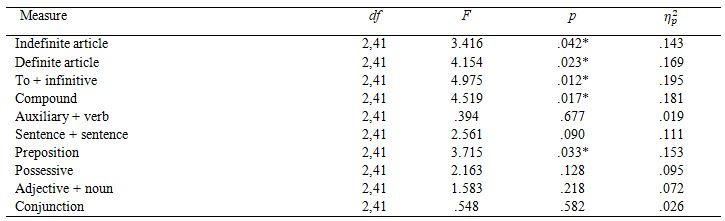
ANOVA results for between-subject effects in mean fixation duration in Experiment 1.

#### Revisits

To see whether NSS subtitles induced more re-reading, which would show their lower readability, we analysed the number of revisits to the subtitles. We found a main effect of segmentation on revisits in all linguistic units apart from *SentSent*, *Prep* and *Conj* (see Table 7). Contrary to expectations, the number of revisits was higher in the SS condition for noun phrases (*IndArt*, *DefArt*, *Comp*, *Poss*). As for verb phrases (*ToInf*, *AuxVerb*) and *AdjN*, revisits were higher in the NSS condition.

We found interactions between segmentation and language in *Poss*, *F*(2,53)=3.418, *p*=.040, η_p_²=.114, and *AdjN,* *F*(2,53)=7.696, *p*=.001, η_p_²=.225. We decomposed these interactions with simple effects with Bonferroni correction and found that for English participants there was a main effect of segmentation on revisits in *Poss*, *F*(1,17)=20.823, *p*=.000, η_p_²=.551, and *AdjN*, *F*(1,17)=5,017, *p*=.039, η_p_²=.228. *Poss* was higher in the SS condition and *AdjN* was higher in the NSS condition. For Polish participants, there was no main effect of segmentation in *Poss*, but there was a main effect in *AdjN*, *F*(1,15)=26.340, *p*=.000, η_p_²=.637, being higher in the NSS condition. For Spanish participants, we found a main effect in *Poss*, *F*(1,21)=5.469, *p*=.029, η_p_²=.207, but only a tendency in *AdjN*, *F*(1,21)=3.980, *p*=.059, η_p_²=.159. They had more revisits for *Poss* in the SS condition, whereas there were more revisits for *AdjN* in the NSS condition.

There was no main effect of language on revisits in any of the linguistic units, apart from *AuxVerb,* *F*(2,53)=6.437, *p*=.003, η_p_²=.195. Post-hoc Bonferroni tests showed that Polish participants made significantly more revisits than Spanish participants, *p*=.003, 95% CI [.37, 2.10], being higher in the NSS for both groups.

**Table 7.**
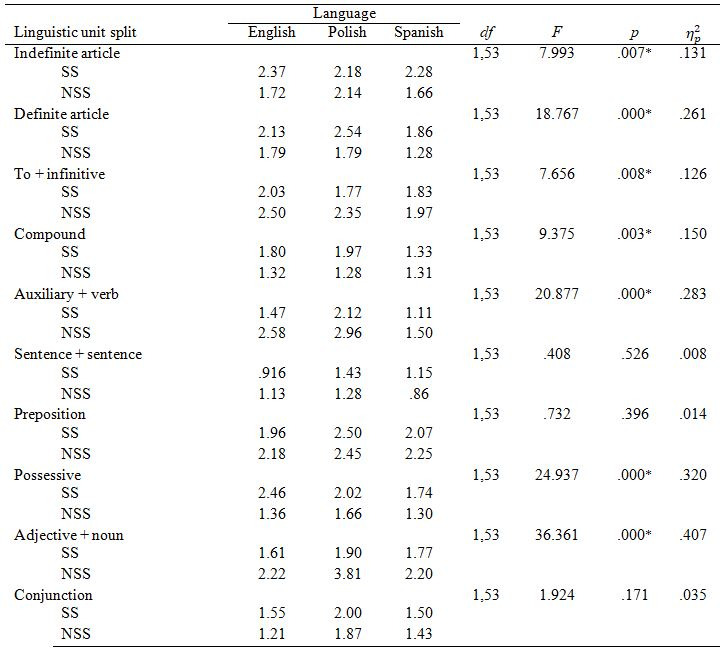
Revisits by linguistic unit and segmentation.

#### Discussion

All participants preferred SS than NSS subtitles. The strongest effect was found in the SS *SentSent* condition, with 86% participants expressing preference for the syntactically cued subtitles compared to 14% for non-syntactically cued ones. Most participants stated they prefer subtitles to be segmented according to semantic and syntactic phrase structures, and not shape.

Two interesting patterns emerged from eye tracking results on the time spent reading the noun and verb phrases in the subtitles. SS subtitles consistently induced longer dwell time for noun phrases (*IndArt, DefArt, Comp, Poss*), whereas NSS subtitles induced longer dwell time for verb phrases (*AuxVerb* and *ToInf*). We observed an interaction effect in English participants: for *Poss*, they had longer dwell time in the SS condition than Spanish and Polish participants.

Results in revisits followed the same pattern: participants made more revisits in the SS subtitles in noun phrases (*IndArt*, *DefArt*, *Comp*, *Poss*) and more revisits in NSS subtitles in verb phrases (*ToInf*, *AuxVerb*). The interactions indicated that there were more revisits for *Adj* in the SS condition across the three groups and for *Poss* in the SS condition for English and Spanish participants. These results seem to indicate that noun phrases are more difficult to process in SS condition, and verb phrases in the NSS condition.

In line with our predictions, Spanish participants, who come from dubbing tradition, showed longer mean fixation duration than English and Polish participants in both SS and NSS subtitles. There was an interaction showing that Polish had more difficulties processing *DefArt* in the NSS condition, with longer mean fixation duration.

### Experiment 2

### Preferences

Similarly, to Experiment 1, we conducted a 2 x 3 mixed ANOVA with segmentation (SS vs. NSS subtitles) as a within-subject factor and hearing loss (hearing, hard of hearing, and deaf) as a between-subjects factor with a percentage of preference for a linguistic unit as a dependent variable.

**Figure 5.**
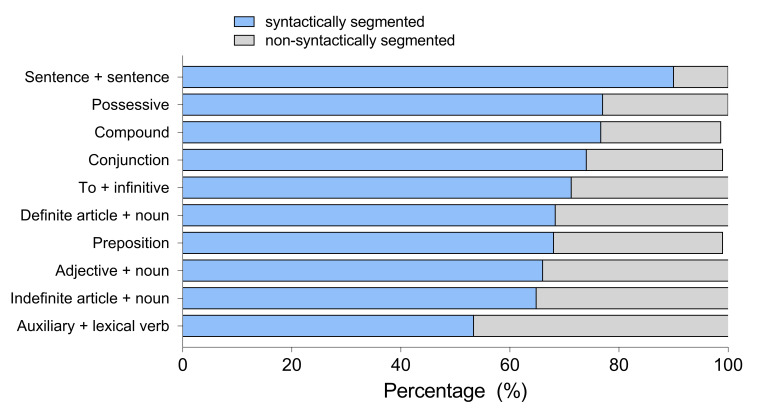
Preferences for SS and NSS subtitles by linguistic units in Experiment 2. Table 8. Percentage of Experiment 2 participants who preferred the syntactically segmented condition.

This time we found a main effect of segmentation in all linguistic parameters apart from *AuxVerb* and *AdjN*: the SS subtitles were preferred over the NSS ones. Figure 5 presents general preferences for all linguistic units and Table 8 shows how they differed by hearing loss.

**Table 8.**
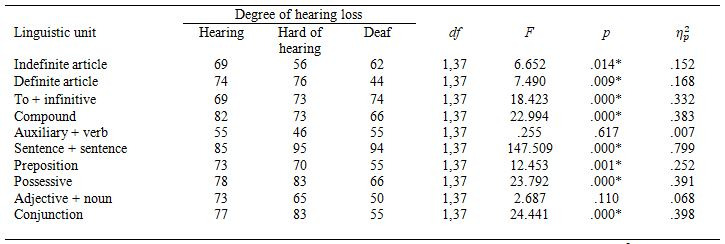
Percentage of Experiment 2 participants who preferred the syntactically segmented condition.

We found an almost significant interaction between segmentation and hearing loss in *DefArt*, *F*(2,37)=3.086, *p*=.058, η_p_²=.143. We decomposed it with simple effects with Bonferroni correction and found that for hearing participants there was a main effect of preference on segmentation in *DefArt*, *F*(1,20)=19,375, *p*=.000, η_p_²=.492, as well as for hard of hearing participants, *F*(1,9)=7.111, *p*=.026, η_p_²=.441, but there was no effect for deaf participants. This means that deaf participants expressed a slight preference towards NSS, but it was not significant.

There was a main effect of hearing loss in AdjN, *F*(2,37)=3.469, *p*=.042, η_p_²=.158 and a tendency approaching significance in Comp, *F*(2,37)=3.063, *p*=.059, η_p_² =.142. Post-hoc Bonferroni tests showed that hearing participants tended to express higher preference for SS *AdjN* than hard of hearing participants, *p*=.051, 95% CI [-.0009, .0834], as well as for SS *Comp*, *p*=.057, 95% CI [-.1001, .0001]. No statistically significant difference was reached in the group of deaf participants.

**Figure 6.**
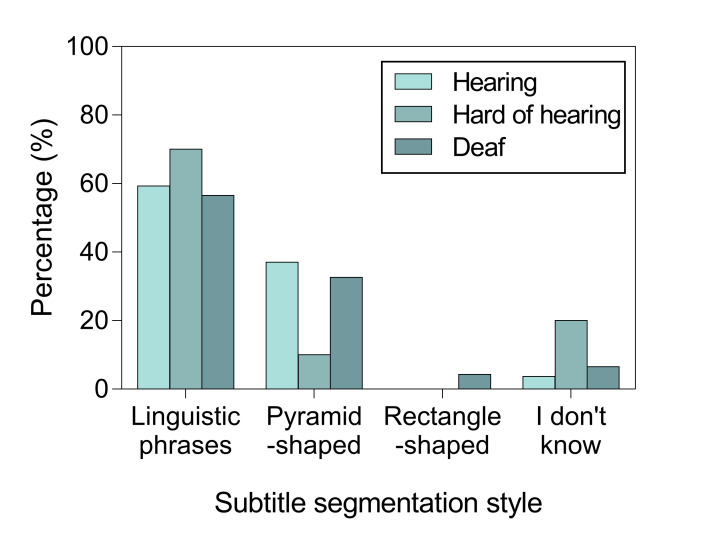
Segmentation preferences by group.

When asked about their choices, most hearing and hard of hearing participants declared to prioritize semantic and syntactic units, whereas for deaf participants it was the subtitle shape that was more important, as shown on Figure 6.

### Eye tracking measures

Due to data quality issues, eye tracking analyses in Experiment 2 were conducted on 16 English, 8 hard of hearing and 5 deaf participants.

#### Dwell time

We found a significant main effect of segmentation on dwell time in *IndArt*, *AuxVerb* and *Poss* (see Table 9). Dwell time was higher for *IndArt* in the SS condition and for *AuxVerb* in the NSS condition.

We found interactions between segmentation and hearing loss in dwell time for *AdjN*, *F*(2,26)= 7.898, *p*=.002, η_p_² =.378, and *Conj*, *F*(2,26)= 4.334, *p*=.024, η_p_² =.250. We decomposed these interactions with simple effects with Bonferroni correction and found that for hard of hearing participants there was a main effect of segmentation on dwell time in *AdjN*, *F*(1,7)=31.727, *p*=.001, η_p_² =.819, and *Conj*, *F*(1,7)=8,306, *p*=.024, η_p_² =.543. Dwell time was higher for *AdjN* in the NSS condition and for *Conj* in the SS condition. Main effect of segmentation of *Poss* for hard of hearing was higher in the SS condition. As for deaf participants, the main effect of segmentation on dwell time for *Poss* was higher in the NSS condition. There was no effect for hearing or deaf participants in *AdjN* and *Conj*.

Between-subject analysis showed a significant main effect of hearing loss in *DefArt* (*F*(2,26)=3.846, *p*=.034, η_p_² = .228) and a tendency approaching significance in *SentSent* (*F*(2,26)=3.241, *p*=.055, η_p_²=.200). Post-hoc tests with Bonferroni correction showed that deaf participants had significantly lower dwell time than hard of hearing in *DefArt*, *p*=.032, 95% CI [-1801.76, -64.33]. Hard of hearing participants tended to have higher dwell time than hearing participants in *SentSent*, *p*=.053, 95% CI [-962.76, -4.14].

**Table 9.**
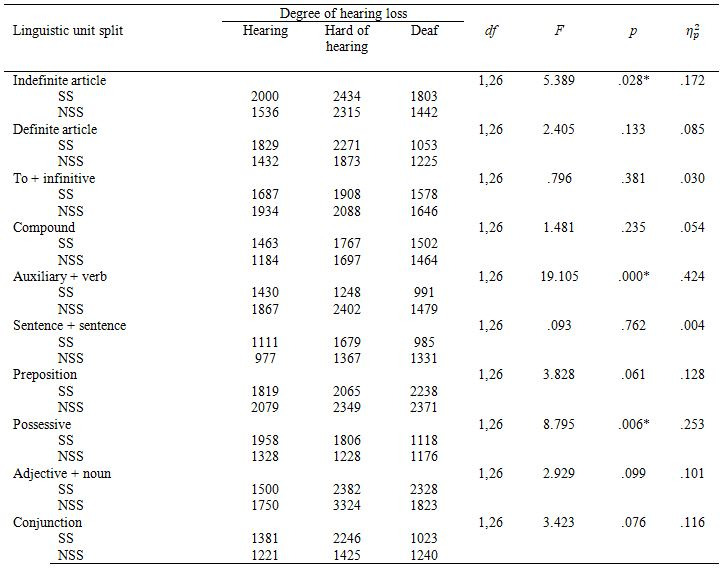
Dwell Time by linguistic unit and segmentation (ms).

#### Mean fixation duration (MFD

Segmentation had no effect on MFD (Table 10) and there were no interactions between segmentation and degree of hearing loss.

There was a main effect of hearing loss on mean fixation duration in *SentSent*, *F*(2,20)=3.603, *p=.*046, η_p_² =.265.

Post-hoc Bonferriori tests showed that hard of hearing participants had significantly longer mean fixation durations than hearing participants in *SentSent*, *p*=.044, 95% CI [-59.84, -64]. Mean fixation duration for *SentSent* was higher in the SS condition for both groups.

**Table 10.**
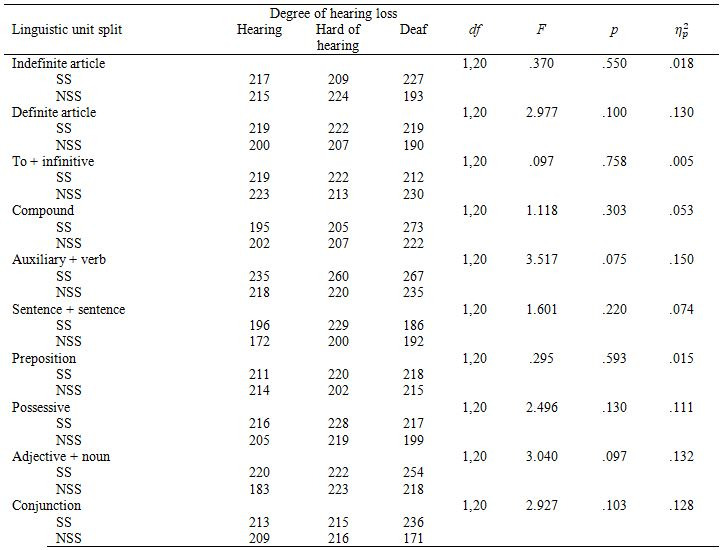
Mean Fixation Duration by linguistic unit and segmentation

#### Revisits

We found a significant main effect of segmentation on revisits in *IndArt*, *AuxVerb* and *Poss*. The number of revisits was higher for *IndArt* and *Poss* in the SS condition and for *AuxVerb* in the NSS condition.

We also found interactions between segmentation and hearing loss in revisits in *ToInf*, *F*(2,29)= 41.48, *p*=.026, η_p_²=.222. We decomposed these interactions with simple effects with Bonferroni correction and found that deaf participants tended to have more revisits for *ToInf* in the SS condition *F*(1,4)=6.968, *p*=.058, η_p_²=.635. There was no effect for English or hard of hearing participants.

**Table 11.**
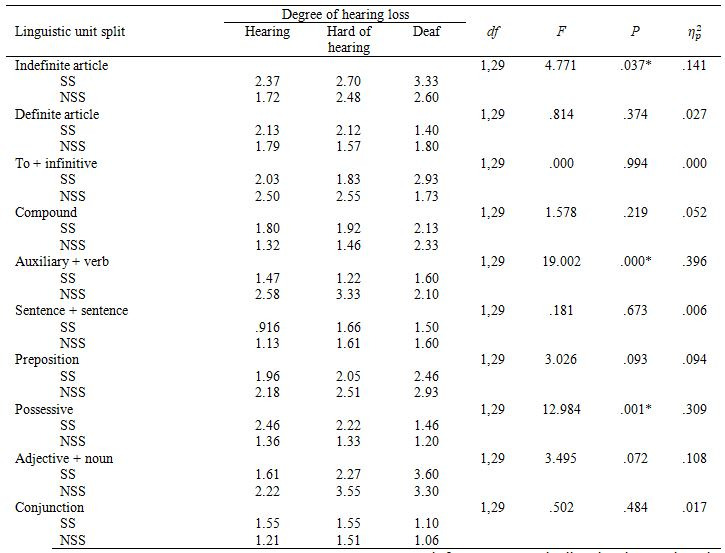
Revisits by linguistic unit and segmentation.n

#### Discussion

Similarly to Experiment 1, most participants expressed a marked preference towards SS subtitles. Again, the strongest effect was in *SentSent* cases with 90% for the SS condition compared to 10% for NSS. Deaf participants showed lower preferences than the other groups for SS subtitles in function words, such as *DefArt*, *Conj*, *Poss* and *Prep*.

Hearing and hard of hearing participants stated clearly they chose subtitles based on semantic and syntactic phrases, whereas deaf participants based their decisions on shape, with the preference towards the pyramid-shaped subtitles.

Deaf participants seemed to have more difficulties processing definite and indefinite articles than the other groups, as shown by eye tracking results: they tended to have more revisits for the SS *ToInf* compared to hearing and hard of hearing participants.

#### Interviews

In the post-task interviews, more than half of the participants of all the groups stated that they preferred line breaks that follow syntactic and semantic rules. However, a number of participants opted for non-syntactic line breaks, stating they give them a sense of continuity in reading, especially for some linguistic categories such as *ToInf* or *IndArt*. Many participants commented that segmentation should keep syntax and shape in balance; subtitles should be chunked according to natural thoughts, so that they can be read as quickly as possible. Other participants specified that segmentation might be an important aspect for slow readers. One interesting observation by a hard of hearing participant was that “line breaks have their value, yet when you are reading fast most of the time it becomes less relevant.”

## General discussion

In this study we investigated the preferences and reactions of viewers to syntactically segmented (SS) and non-syntactically segmented (NSS) text in subtitles. Our study combined an offline, metalinguistic measure of preference with online eye tracking-based reading time measures. To determine whether these measures depend on previous experience with subtitling or on hearing loss, we tested participants from countries with different audiovisual translation traditions: hearing people from the UK, Poland and Spain as well as British deaf, hard of hearing, and hearing viewers. We expected participants to prefer SS subtitles as this type of segmentation follows the “natural sentence structure” (21). We also hypothesized that NSS text would be more difficult to read, resulting in longer reading times. Our predictions were confirmed in relation to preferences, but only partially confirmed when it comes to eye tracking measures.

The most important finding of this study is that viewers expressed a very clear preference for syntactically segmented text in subtitles. They also declared in post-test interviews that when making their decisions, they relied more on syntactic and semantic considerations rather than on subtitle shape. These results confirm previous conjectures expressed in subtitling guidelines (5, 6) and provide empirical evidence in their support.

SS text was preferred over NSS in nearly all linguistic units by all types of viewers except for the deaf in the case of the definite article. The largest preference for SS was found in the *SentSent* condition, whereas the lowest in the case of *AuxVerb*. The *SentSent* condition was the only one in our study which included punctuation. The two sentences in a subtitle were clearly separated by a full stop, thus providing participants with guidance on where one unit of meaning finished and another began. Viewers preferred punctuation marks to be placed at the end of the first line and not separating the subject from the predicate in the second sentence, thus supporting the view that each subtitle line should contain one clause or sentence (6). In contrast, in the *AuxVerb* condition, which tested the splitting of the auxiliary from the main verb in a two-constituent verb phrase, the viewers preferred SS text, but their preference was not as strong as in the case of the *SentSent* condition. It is plausible that in order to fully integrate the meaning of text in the subtitle, viewers needed to process not only the verb phrase itself (auxiliary + main verb), but also the verb complement.

Contrary to our predictions, some linguistic units took longer to read in the SS rather than NSS condition, as reflected by longer dwell time and more revisits. To interpret the differences between linguistic units, we classified some of them as noun or verb phrases. The *IndArt*, *DefArt*, *Comp* and *Poss* conditions were grouped under the umbrella term ‘noun phrases’, whereas *AuxVerb* as ‘verb phrases’. In general, people spent more time reading the SS text in noun phrases, and less time reading the NSS text in the *AuxVerb*. This finding goes against the results reported by (27), who tested ‘ill-segmented’ and ‘well-segmented’ noun phrases in Italian subtitles on a group of hearing people, and found no differences in the number of fixations or proportion of fixation time between the SS and NSS conditions. Interestingly, the authors also found a slightly longer mean fixation duration on NSS subtitles (228 ms in NSS compared to 216 ms in SS) – a result which was not confirmed by our data. In fact, in our study the mean fixation duration in the noun phrase *AdjN* in Experiment 1 was longer in the SS than in the NSS condition. That readers looked longer at this noun phrase category in the SS condition may be attributed to its final position at the end of the first subtitle line.

Compare, for instance:

 (SS) He's looking for the memory stick

 he managed to hide.

and

 (SS) He's looking for the memory 

stick he managed to hide.

where in the SS condition, the complete noun phrase *Comp* is situated at the end of the first subtitle line. (64) found that readers looked longer at noun phrases when they were in the clause-final position. Syntactically segmented text in subtitles is characterized by the presence of complete phrases at the end of lines (6). According to Rayner et al. (64), readers “fixate longer on a word when it ends a clause than when the same word does not end a clause,” which could explain the longer fixation time. This result may be taken as an indication that people integrate the information from the clause at its end, including any unfinished processing before they move on, which has been referred to in literature as “clause wrap-up effect” (64, 65).

This study also brought to light some important difference between how various types of viewers process line breaks in subtitling. Spanish viewers, who are generally less accustomed to subtitling and more to dubbing, had longest mean fixation duration in a number of linguistic units, indicating more effortful cognitive processing (60) compared to Polish participants, who were more accustomed to subtitling. This result is not necessarily related to the nature of text segmentation, but rather to participant characteristics.

We also discovered interesting patterns of results depending on hearing loss. Deaf participants were not as concerned about syntactic segmentation as other groups, which was demonstrated by a lack of effect of segmentation on preferences in some linguistic units. This finding confirms our initial prediction about deaf people experiencing more difficulties in processing syntactic structures. The fact that there was no effect of segmentation in *DefArt* for deaf participants, combined with their longer dwell time spent on reading sentences in the *DefArt* condition, should perhaps be unsurprising, considering that deaf people with profound and severe prelingual hearing loss tend to experience difficulties with function words, including articles (48, 49, 50). This effect can be attributed to the absence of many function words in sign languages, their context-dependence and low fixed semantic content (48, 66).

One important limitation of this study is that we tested static text of subtitles rather than dynamically changing subtitles displayed naturally as part of a film. The reason for this was that this approach enabled us to control linguistic units and to present participants with two clear conditions to compare. However, this self-paced reading allowed participants to take as much time as they needed to complete the task, whereas in real-life subtitling, viewers have no control over the presentation speed and have thus less time to process subtitles. The understanding of subtitled text is also context-sensitive, and as our study only contained screenshots, it did not allow participants to rely more on the context to interpret the sentences, as they would normally do when watching subtitled videos. Another limitation is the lack of sound, which could have given more context to hearing and hard of hearing participants. Yet, despite these limitations in ecological validity, we believe that this study contributes to our understanding of processing different linguistic units in subtitles.

Future research could look into subtitle segmentation in subtitled videos (see also Gerber-Morón and Szarkowska (28)), using other languages with other syntactic structures than English, which was the only language tested in this study. Further research is also required to fully understand the impact of word frequency and word length on the reading of subtitles (67, 68). Subtitle segmentation implications could also be explored across subtitles, when a sentence runs over two or more subtitles.

Our findings may have direct implications on current subtitling practices: if possible, text in the subtitles should be segmented to keep syntactic phrases together. This is particularly important in the case of two clauses or sentences separated by a punctuation mark. It is perhaps less important in the case of verb phrases like auxiliary and main verb. Following syntactic rules for segmenting subtitles can facilitate the reading process to viewers less experienced with subtitling, and can benefit deaf viewers from improving their syntax.

### Ethics and Conflict of Interest

The authors declare that the contents of the article are in agreement with the ethics described in http://biblio.unibe.ch/portale/elibrary/BOP/jemr/ethics.html and that there is no conflict of interest regarding the publication of this paper.

### Acknowledgements

The research reported here has been supported by a grant from the European Union’s Horizon 2020 research and innovation programme under the Marie Skłodowska-Curie Grant Agreement No. 702606, “La Caixa” Foundation (E-08-2014-1306365) and Transmedia Catalonia Research Group (SGR2005/230).

Many thanks for Pilar Orero and Judit Castellà for their comments on an earlier version of the manuscript.
